# Endogenous small interfering RNAs associated with maize embryonic callus formation

**DOI:** 10.1371/journal.pone.0180567

**Published:** 2017-07-03

**Authors:** Fei Ge, Xing Huang, Hongmei Hu, Yanling Zhang, Zhaoling Li, Chaoying Zou, Huanwei Peng, Lujiang Li, Shibin Gao, Guangtang Pan, Yaou Shen

**Affiliations:** 1 Key Laboratory of Biology and Genetic Improvement of Maize in Southwest Region, Maize Research Institute of Sichuan Agricultural University, Chengdu, Sichuan Province, China; 2 Institute of Animal Nutrition, Sichuan Agricultural University, Chengdu, Sichuan Province, China; Huazhong University of Science and Technology, CHINA

## Abstract

The induction efficiency of maize embryonic callus is highly dependent on the genotype, and only a few lines possess a high capacity for callus formation. Although certain genes and pathways have been reported to contribute to the regulation of callus induction, to the best of our knowledge, the functions of the small interfering RNAs (siRNAs) involved in this process remain unknown. In this study, we identified 861 differentially expressed siRNAs and 576 target genes in the callus induction process. These target genes were classified into 3 clusters, and their functions involve controlling metalloexopeptidase activity, catalase activity, transcription regulation, and O-methyltransferase activity. In addition, certain genes related to auxin transport and stem cell or meristem development (e.g., *PLT5-like*, *ARF15*, *SAUR-like*, *FAS1-like*, *Fea3*, *SCL5*, and *Zmwox2A*) were regulated by the differentially expressed siRNAs. Moreover, zma-siR004119-2 directly cleaves the 5’ UTR of *Homeobox-transcription factor 25*, which further leads to the down-regulation of its expression. Twelve 24-nt-siRNAs led to the hyper-methylation of GRMZM2G013465, which further decreases its expression. These results suggest that differentially expressed siRNAs regulate callus formation by controlling the expression of their target genes.

## Introduction

Transgenic maize (*Zea mays* L.) is currently widely used to study gene functions and breeding. Maize embryonic calli that are induced from immature embryos are the most important explants for transformation. However, only a few maize lines have a high frequency of embryonic callus induction [1,2]. According to a previous study, the dedifferentiation efficiency is determined by the genotype [3]. Previous studies using RNA-seq [2], small RNA-seq [4] and proteomic analyses of maize embryonic calli [5–7] have revealed that most differentially expressed genes are involved in many processes, such as regulating pyruvate biosynthesis [6], hormone transduction [7], stress response [5], and cell proliferation [5]. Recently, several genes have been shown to control embryonic callus induction, including *BABY BOOM* [8], the *WUSCHEL*-*CLAVATA3* feedback loop [8,9], and *LEAFY COTYLEDON* [10].

In addition, many small non-coding RNAs have been shown to be involved in somatic embryogenesis in cotton [11], poplar [12] and citrus [13]. Small non-coding RNAs, which are classified as microRNAs (miRNAs) and small interfering RNAs (siRNAs), play important roles in regulating gene expression through transcriptional and posttranscriptional gene silencing in plants [14–17]. Most miRNAs have been reported to regulate plant development and respond to biotic and abiotic stress [18–20]. We previously identified 21 differentially expressed miRNA families during embryonic callus formation in the maize inbred line 18-599R that target 87 genes, resulting in the regulation of plant hormone signal transduction, ECM-receptor interaction, antigen processing and presentation, and alpha-linolenic acid metabolism pathways [4]. Similarly, 36 differentially expressed miRNA families and 50 differentially expressed miRNAs were reported to be responsible for cotton [11] and citrus [13] somatic embryogenesis, respectively.

Furthermore, siRNAs control plant growth and development by negatively regulating the expression level of target genes to repress their function. In plants, siRNAs have been categorized into several classes containing repeat-associated siRNAs (ra-siRNAs), natural antisense transcript-derived siRNAs (nat-siRNAs), trans-acting siRNAs (ta-siRNAs), heterochromatic siRNAs (hc-siRNAs), secondary transitive siRNAs, and long siRNAs [16,19]. Endogenous siRNAs participate in many biological processes, such as hybrid vigor [15], biotic and abiotic stress responses [16,21], and heterochromatin gene silencing [17].

Moreover, 4 tas3-siRNAs that are derived from the miRNA390-mediated cleavage of their precursors were reported to target to 2 *ARF* genes to potentially promote cotton somatic embryogenesis [11]. Similarly, most of the 459 differentially expressed siRNAs between the citrus embryonic callus and the non-embryonic callus were down-regulated, resulting in the activation of their target genes, which further regulates the stress-response process and other cell differentiation biological processes [13]. We hypothesize that siRNAs may play important roles in the dedifferentiation of maize immature embryos. Therefore, we re-analyzed our previous deep sequencing data of small RNAs from embryonic calli to identify differentially expressed siRNAs and identify their potential roles in controlling callus induction [4].

## Materials and methods

### Samples preparation, RNA isolation, and real-time qPCR

The immature embryo of the maize inbred line 18-599R (18R), which was provided by the Maize Research Institute of Sichuan Agricultural University, possesses a high embryonic callus induction efficiency, and we therefore used this line to study the potential role of siRNAs in embryonic callus formation. Each sample consisted of 1 g of embryos or calli that were induced for 0–15 d, and then the total RNA was isolated from each sample using TRIzol Reagent (Invitrogen, Carlsbad, CA 92008, USA) according to the manufacturer’s instructions. The embryonic callus formation process is classified into the following 3 primary stages according to the phenotypic characteristics: Stage I, embryo intumescence period (induced for 1–5 d); Stage II, initial callus formation (induced for 6–10 d); and Stage III, embryonic callus formation (induced for 11–15 d). Thus, 10 μg of RNA from the samples at 1–5 d were mixed to yield the Stage I sample; 10 μg of RNA from the samples at 6–10 d were mixed to produce the Stage II sample; and 10 μg of RNA from the samples at 11–15 d were mixed to generate the Stage III sample. The control sample (CK) consisted of 10 μg of RNA isolated from immature embryos that were not induced (0 d). The construction of the small RNA libraries and the sequencing were performed as described in our previous study [4].

The cDNAs from the CK, Stage I, Stage II and Stage III samples used in the real-time qPCR analysis of the siRNAs and their target genes were reverse transcribed using the “Mir-X^™^ miRNA First-Strand Synthesis Kit” (Clontech, Dalian, China) and the “PrimeScript RT Reagent Kit with gDNA Eraser” (Takara, Dalian, China), respectively, according to the manufacturers’ instructions. We performed qPCR on an ABI 7500 real-time PCR System (Applied Biosystems, USA). *Actin 1* (GRMZM2G126010) and 5S (gi:114151623) were used as endogenous controls for quantifying the target gene and siRNA expression levels, respectively. All primers are listed in S3 Table. To verify the significance of the DE-siRNAs and target genes, we performed real-time qPCR using a few lines with different embryonic callus formation efficiencies. Here, 3 lines (i.e., IBM107, IBM127 and IBM164) with a low embryonic callus induction rate were analyzed and compared to 18R.

### Identification of siRNAs

After the removal of adapter sequences and low-quality reads, the small RNA reads were mapped to the maize B73 genome v3 (RefGen_v3). Only perfect matches were included in further analyses. Then, the rRNA, snRNA, snoRNA, tRNA and miRNA sequences were removed. The remaining small RNAs were submitted to a siRNA prediction based on the following two principles: (i) 2 sequences are complemented and have two overhanging bases and (ii) either of the 2 sequences has more than 5 reads in any of the samples.

### Identification of differentially expressed siRNAs

The siRNA reads were normalized by converting the reads into reads per million clean reads (RPM). Only the siRNAs with *P* < 0.01 and an absolute value of log_2_ (siRNA RPM in stages/control) ≥ 1 were considered differentially expressed siRNAs (DE-siRNAs) during embryonic callus formation. The *P-value* used in the comparison of the DE-siRNAs was calculated using the following formula [22]: $P\left(\text{x}|\text{y}\right)={\left(\frac{\text{N}2}{\text{N}1}\right)}^{\text{y}}\frac{\left(\text{x}+\text{y}\right)!}{\text{x}!\text{y}!{\left(1+\frac{\text{N}2}{\text{N}1}\right)}^{\left(\text{x}+\text{y}+1\right)}}$. N1 and N2 represent the total number of reads in the two compared samples, and x and y represent the numbers of siRNA reads in the two samples.

### Prediction of the target genes of the DE-siRNAs

TargetFinder (http://targetfinder.org/index.php/home) and psRobot (http://omicslab.genetics.ac.cn/psRobot/) were used to predict the target genes of all differentially expressed siRNAs. In this study, the target genes whose sense strand was targeted by the differentially expressed siRNAs (DE-siRNAs) but not by 24-nt siRNAs were included in further analyses. By combining these results with our previous digital gene expression (DGE) profiling data of tissue samples from the same stages [2], only the target genes that were differentially expressed [Log_2_(fold change) > 1, FDR < 0.001] and showed negative correlations with the siRNAs (correlation < -0.4) were maintaining in the analysis. Additionally, 24-nt siRNAs have been reported to trigger DNA methylation, which further leads to the transcriptional silencing of the target genes [23]. Therefore, the 24-nt DE-siRNAs that target both the sense and antisense strands of the target genes were also included in further analyses. By combining our DNA methylation data [24] and the DGE profiling data [2] of tissue samples from the same stages, further analyses included those target genes that showed changes in their expression levels and were negatively correlated with the changes in their DNA methylation levels (Benjamini-Hochberg *P-value* < 0.05, mean signal in at least one sample > 0.25 reads per million, and the ratio between CK and the other stage > 2 were considered significantly changed) and the expression levels of the corresponding 24-nt DE-siRNAs. All target genes identified above were submitted to a K-means cluster analysis using “ExpressCluster v1.3” software (http://gparc.org/view/urn:lsid:8080.SD148.127.0.0.1:genepatternmodules:19:1.3). Finally, we performed GO enrichment analyses for each cluster using the OmicShare tool (www.omicshare.com/tools).

## Results and discussion

### siRNAs involved in the induction of immature embryonic calli

**Fig 1:**
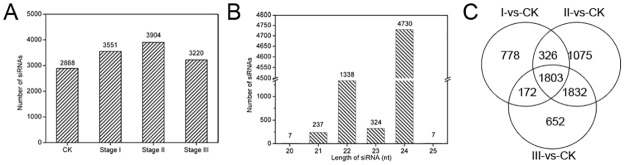
siRNAs identified in the process of callus formation. (A) The number of siRNAs identified in the 4 samples. (B) The number of siRNAs of different lengths. (C) Venn diagram of the differentially expressed siRNAs identified in the 4 samples.

The small RNA deep sequencing data were obtained from our previous study [4]. After removing the low-quality sequences, adapter sequences, rRNA, snRNA, snoRNA, tRNA, microRNAs, and size smaller than 18 nt, we identified 2888, 3551, 3904, and 3220 siRNAs in CK, Stage I, Stage II and Stage III, respectively (Fig 1A). In this study, the length distribution of the siRNAs ranged from 20 to 25 nucleotides (nt), which is consistent with the lengths observed in other plants [25,26], and the 24- and 22-nt siRNAs are the most and second most abundant siRNAs in the 4 samples (Fig 1B).

To identify the important siRNAs that are involved in callus induction, we compared the expression level in each stage to that in CK to analyze the DE-siRNAs. Only the siRNAs with fold changes > 2 and *P*-*values* < 0.01 were considered DE-siRNAs (S1 Table). As shown in Fig 1C, 778, 1075, and 652 siRNAs were differentially expressed during Stages I, II and III, respectively. Moreover, 326, 172, and 1832 siRNAs were differentially expressed during Stages I and II, Stages I and III, and Stages II and III, respectively, suggesting that these siRNAs may function only in the two corresponding stages.

To verify the results of the deep sequencing, most DE-siRNAs discussed in this paper were selected for a quantitative real-time PCR analysis. The expression levels observed in the samples from Stages I, II and III were compared with those in the control. As expected, a strong correlation (> 0.82, S1 Fig) was revealed between the deep sequencing and quantitative real-time PCR data, suggesting that the deep sequencing data are credible.

### Target genes of the DE-siRNAs

To understand the mechanism by which the siRNAs regulate embryonic callus formation, we performed a target gene prediction of the DE-siRNAs using the programs psRobot and TargetFinder. Only the genes that completely conformed to the following conditions were considered target genes: i) DE-siRNAs that target the sense strand of the gene; ii) log_2_ (fold change of gene) > 1 and FDR < 0.001; and iii) the correlation between the expression level of the DE-siRNA and the predicted target gene was less than -0.4. In total, 553 differentially expressed genes were identified as target genes of 861 DE-siRNAs (S1 Table).

**Fig 2:**
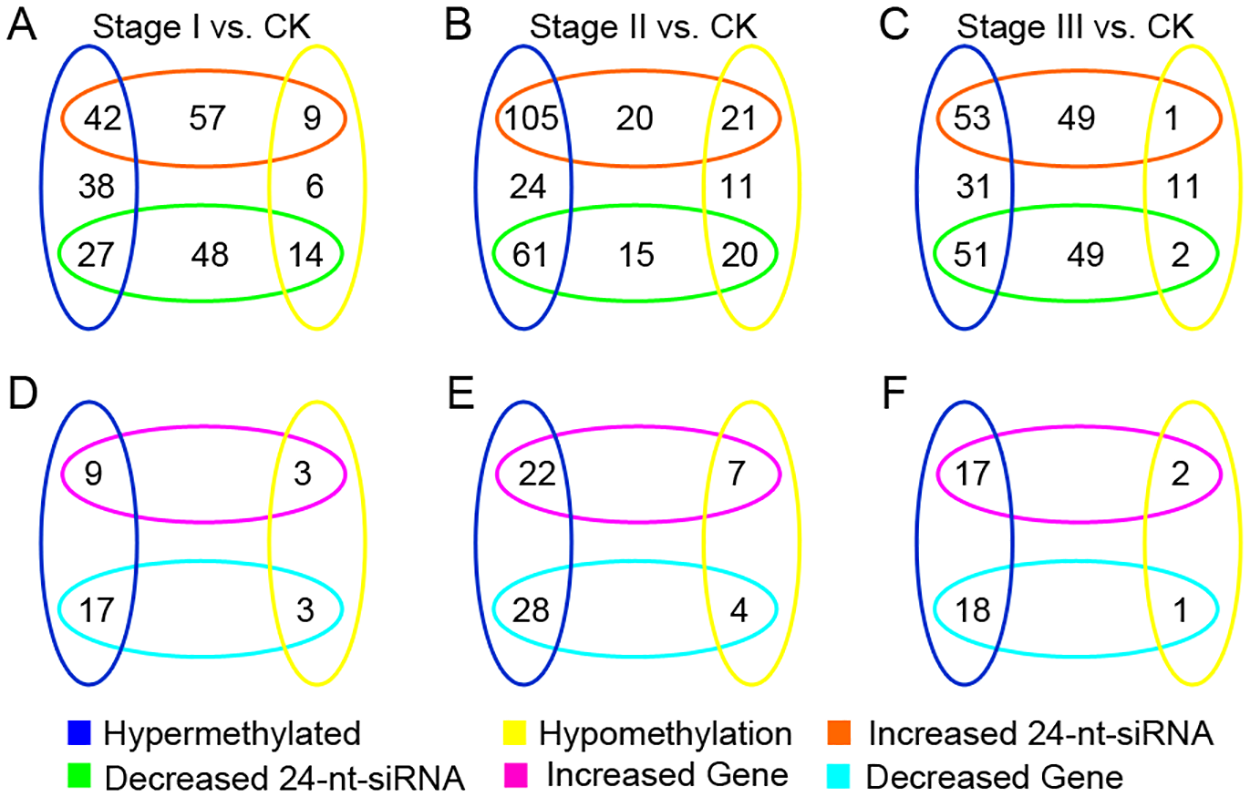
Differentially expressed siRNAs, differentially expressed genes, and differentially methylated genes in embryonic calli. (A-C) Venn diagrams displaying the numbers of differentially expressed siRNAs and differentially methylated target genes in the 3 stages. (D-F) Venn diagrams displaying numbers of differentially expressed and differentially methylated target genes in the 3 stages. The *P*-*values* of the correlations are shown in S2 Table.

In addition, 24-nt siRNAs have the potential to trigger DNA methylation in their target genes [23]. Therefore, the genes that conformed to the following conditions were also identified as target genes: i) the antisense strands were targeted by 24-nt DE-siRNAs, ii) the DNA methylation level was significantly changed (*P* < 0.05), and iii) the expression level was negatively correlated with that of the corresponding 24-nt DE-siRNAs (correlation < -0.4). In this study, compared with CK, 42, 105, and 53 24-nt DE-siRNAs were significantly up-regulated (*P* < 0.01) with hyper-methylation in the corresponding target genes during Stages I, II and III, respectively (Fig 2A–2C). Furthermore, compared with CK, 14, 20, and 2 genes were hypo-methylated with corresponding significant decreases in the 24-nt DE-siRNAs (*P* < 0.01) during Stages I, II and III, respectively (Fig 2A–2C). Furthermore, these genes were used to analyze the correlation between methylation and the gene expression levels. As shown in Fig 2D–2F, compared with CK, 17, 28, and 18 hyper-methylated genes were down-regulated during Stages I, II and III, and 3, 7, and 2 hypo-methylated genes were up-regulated during Stages I, II and III, respectively. Finally, 36 target genes whose antisense strands were bound by 24-nt DE-siRNAs were identified. Importantly, in 13 target genes, both the sense and antisense strands were targeted by 24-nt DE-siRNAs. In total, we identified 576 potential target genes for all DE-siRNAs.

### GO analysis of the target genes of DE-siRNAs

**Fig 3:**
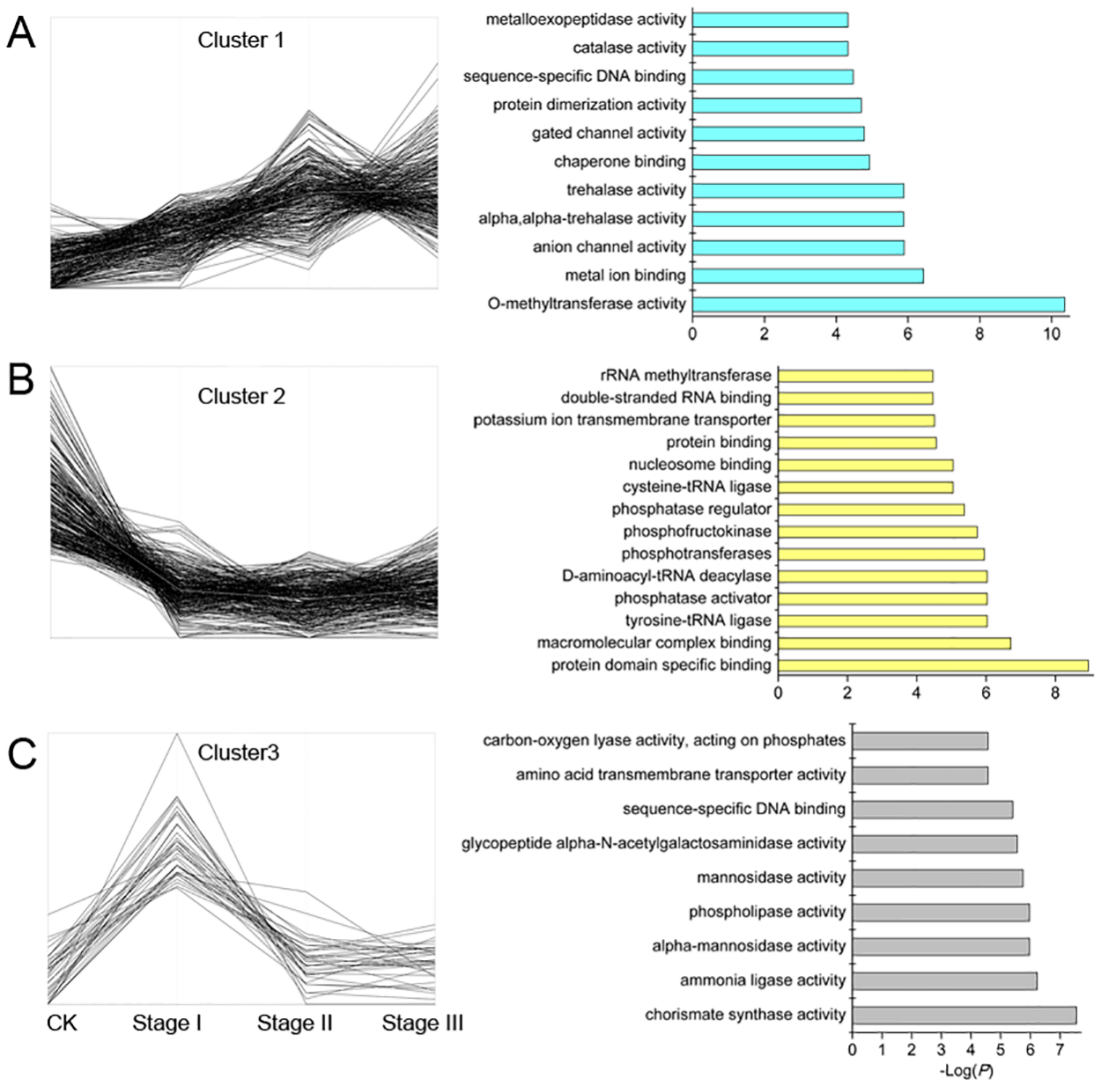
Expression patterns and gene functions of target genes. (A-C) The patterns of target genes and significantly enriched (*P* < 0.05) GO terms in clusters 1, 2 and 3.

To analyze the potential role of the DE-siRNAs in the callus induction process, the 576 target genes were then submitted to cluster analyses (K-means approach) and GO enrichment analyses (http://www.omicshare.com/tools/index.php/). These target genes were classified into 3 clusters (Fig 3).

The target genes in cluster 1 were significantly up-regulated (*FDR* < 0.001) during Stage I and maintained an upward trend in all stages, suggesting that these target genes function as rapid and continuous response factors in the embryonic callus induction process. As shown in Fig 3A, the genes in cluster 1 were significantly enriched in catalase activity, sequence-specific DNA binding, protein dimerization activity, gated channel activity, chaperone binding, trehalase activity, anion channel activity, metal ion binding, and O-methyltransferase activity. As illustrated above, numerous genes were hyper-methylated during the immature embryo dedifferentiation process. As expected, 4 target genes in cluster 1 were annotated with the function of O-methyltransferase activity, which may indicate that the DE-siRNAs control the expression level of methyl-transferase-related genes, which further trigger the DNA methylation of downstream genes to regulate callus induction. As previously reported, several metal ions used to supplement the induction medium, such as Ca, Fe, Zn, K, and Mg, are coenzymes or cofactors of one enzyme or protein. Thus, the up-regulated genes that are related to metal ion binding may function in metal transportation, storage, or enzyme functioning [27].

In contrast, certain target genes classified into cluster 2 were sharply down-regulated during Stage I and remained at low levels throughout the three stages, illustrating their inhibitory roles in callus induction. Based on these target genes, rRNA methyltransferase, double-stranded RNA binding, potassium ion transmembrane transporter, protein binding, nucleosome binding, cysteine-tRNA ligase, phosphatase regulator, phosphofructokinase, phosphotransferases, and D-aminoacyl-tRNA deacylase activities were significantly enriched (*P* < 0.05, Fig 3B). Most of the GO functions of cluster 2 were associated with transcription regulation activity, reflecting their function in promoting or repressing downstream genes, which down-regulate embryonic callus formation.

However, 43 target genes in cluster 3 were sharply up-regulated during Stage I, followed by a rapid decrease to the original level during Stages II and III. These target genes potentially function as immediate response factors that are required for embryo expansion and cell growth. The GO analyses identified significant enrichment in carbon-oxygen lyase activity, acting on phosphates, amino acid transmembrane transporter activity, sequence-specific DNA binding, glycopeptide alpha-N-acetylgalactosaminidase activity, and mannosidase activity (*P* < 0.05, Fig 3C). During embryonic callus induction in *Vitis vinifera* [27] and *Vanilla planifolia* [28], stress-response-related proteins were identified as differentially expressed. Similarly, in this study, GRMZM2G058491 was annotated with the “cellular response to stimulus” process, which reflects its role in the rapid response to the induction medium (auxin stress) during Stage I (embryo expanding stage). In addition, GRMZM2G420882 was annotated with the process of cell communication, which may play an important role in signal transport to regulate the response to auxin stress in immature embryos.

### Important target genes of DE-siRNAs involved in callus induction

**Fig 4:**
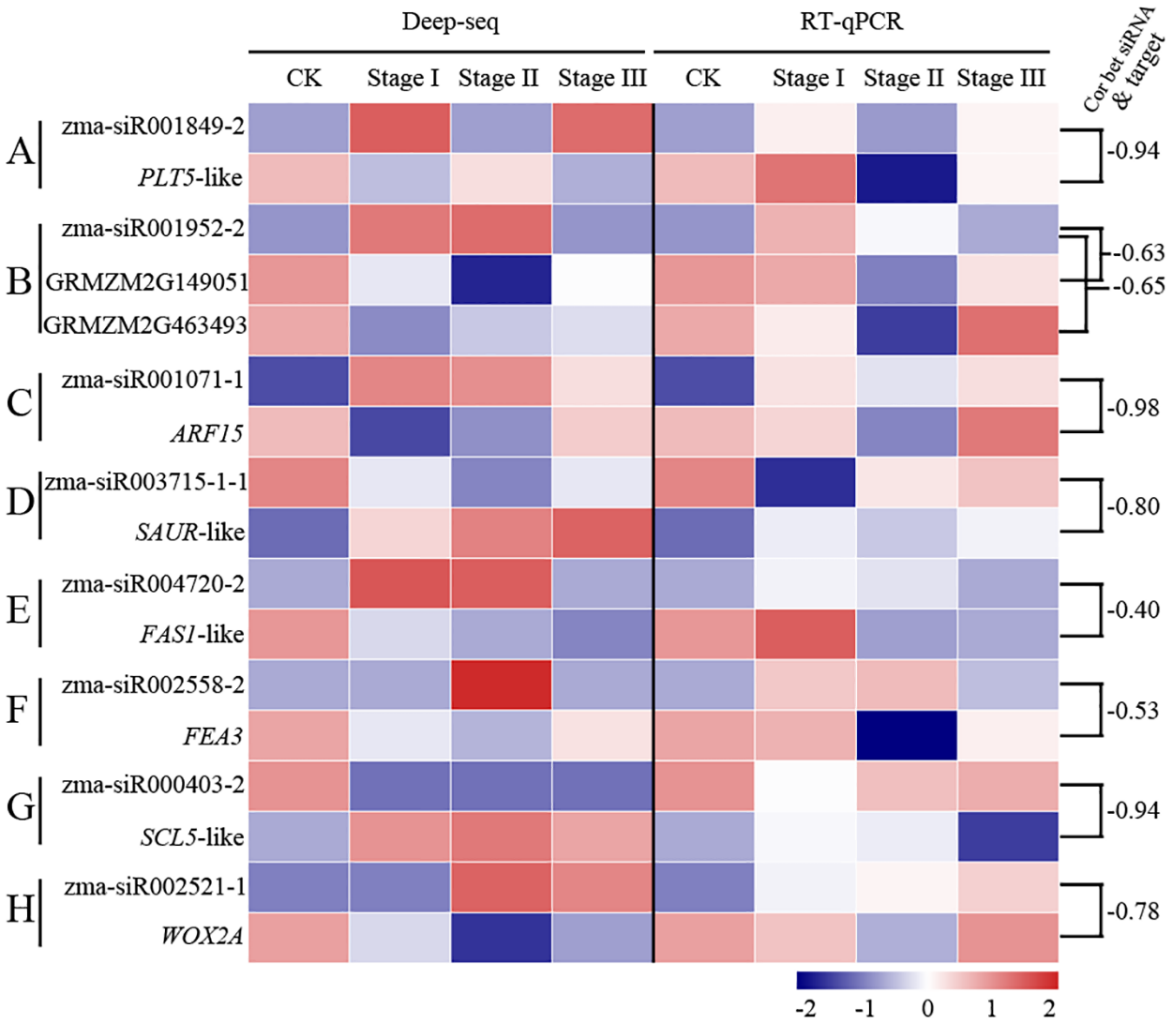
Expression of 8 DE-siRNAs and their target genes in 4 samples. Expression is shown as log_2_ (FC) as calculated in the comparison of the samples from Stages I, II and III to the CK sample. “Deep-seq” and “RT-qPCR” represent the relative expression levels of the siRNAs and target genes derived from the deep sequencing and real-time qPCR, respectively. “cor bet siRNA & target” represent the correlation between the expression level of the siRNA and its target genes derived from the deep sequencing.

Auxin is widely used to induce callus formation in plants and has been shown to play a very important role in cell dedifferentiation [29]. 2,4-D, which is an auxin analog, has been widely used to induce embryonic callus formation [30]. In this study, we found the following three target genes that are related to auxin response and transport: *PLETHORA 5*-*like* (*PLT5-like*, GRMZM2G086573), *ARF15* (GRMZM2G081406) and *SAUR-like* (GRMZM2G442000). As previously reported, the *PLT* genes are required for the establishment and maintenance of stem cell populations, induced by auxin and involved in promoting the expression of *PIN3*, *PIN4* and *PIN7* [31]. It is well known that PIN family proteins (localized on the plasma membrane) actively transport auxin out of cells based on their polar localization, which further regulates cell development [32]. Therefore, the *PLT* genes may influence the polarity of auxin transport by regulating the expression of *PINs*. In this study, zma-siR001849-2 was significantly up-regulated (*P* < 0.01), which may lead to a decreased expression of its target *PLT5-like* gene (Fig 4A) during embryonic callus formation. In addition, GRMZM2G149051 and GRMZM2G463493 were annotated as *RGF1 INSENSITIVE 1*-like genes, which have been reported to establish the gradient of *PLT* genes [31]. Interestingly, GRMZM2G149051 and GRMZM2G463493 were sharply down-regulated during Stages I, II and III (Fig 4B) and were both targeted by zma-siR001952-2. Therefore, we speculate that the *PLT5-like* gene was repressed by zma-siR001849-2, which further regulated auxin transport during immature embryo dedifferentiation in maize.

Similarly, the expression level of *ARF15* decreased in all 3 stages and was negatively correlated with zma-siR001071-1 (-0.98, Fig 4C), reflecting that a balanced auxin signaling transport must also be maintained. The SAUR family proteins are involved in the auxin signaling response that regulates the expression levels of downstream genes. In this study, zma-siR003715-1-1 was down-regulated, which led to an increase in its target gene *SAUR-like* (GRMZM2G442000) during callus formation (Fig 4D); the *SAUR-like* gene then further regulates the expression levels of downstream genes to induce embryonic callus formation.

As shown in Fig 4E and 4F, the *FASCIATA 1-like* (*FAS1 like*, GRMZM2G096458) and *Fasciated Ear3* (*Fea3*, GRMZM2G166524) genes were suppressed by zma-siR004720-2 and zma-siR002558-2, respectively. In Arabidopsis, *FAS1*, *FAS2* and *MSI1* encode *Chromatin Assembly Factor-1* (*CAF-1*), which is considered essential for maintaining gene expression in meristem cells [31,33]. The Arabidopsis shoot apical meristem in the *fas1* and *fas2* mutants were broader and flatter [33], reflecting that the down-regulation of the maize *FAS-like* gene can promote callus induction. In maize, FEA3 was hypothesized to perceive a new CLE (ZmFCP1) signal from the organ primordia to further regulate stem cell activity [34]. The loss of the function of *fea3* results in an enlarged and fasciated meristem, suggesting that the down-regulation of *FEA3* and *FAS1-like* benefits embryonic callus formation.

In this study, GRMZM2G431309, which is an ortholog of the Arabidopsis *scarecrow-like 5* (*SCL5*) gene, was significantly up-regulated (*FDR*< 0.001) and targeted by zma-siR000403-2 (Fig 4G). SCARECROW is required to control the stem cell population and differentiation and maintain the non-cell-autonomous capacity of the quiescent center [35]. It can be speculated that the down-regulation of zma-siR000403-2 results in the up-regulation of *SCL5*, which further maintains the callus non-cell-autonomous capacity and controls the calli population.

The *WUSCHEL* (*WUS*) gene family is well known to function in the induction of the stem cell identity to a non-cell-autonomous capacity [36]. *WUS*, together with *CLV*, forms the well-characterized *CLV*–*WUS* feedback loop, which maintains the stem cell population. Recently, the overexpression of *Baby boom* (*Bbm*) and *Wuschel2* (*Wus2*, GRMZM2G028622) in mature maize seed embryos and leaf segments increased the frequency of embryonic callus formation [8]. However, in this study, *Zmwox2A* (GRMZM2G108933) was down-regulated by zma-siR002521-1 during the callus induction stages (Fig 4H). *Zmwox2A* may participate in controlling callus induction via a different pathway; alternatively, the decrease may be due to the functional redundancy of the *WUSCHEL* genes due to the up-regulation of *WOX5B* [2].

**Fig 5:**
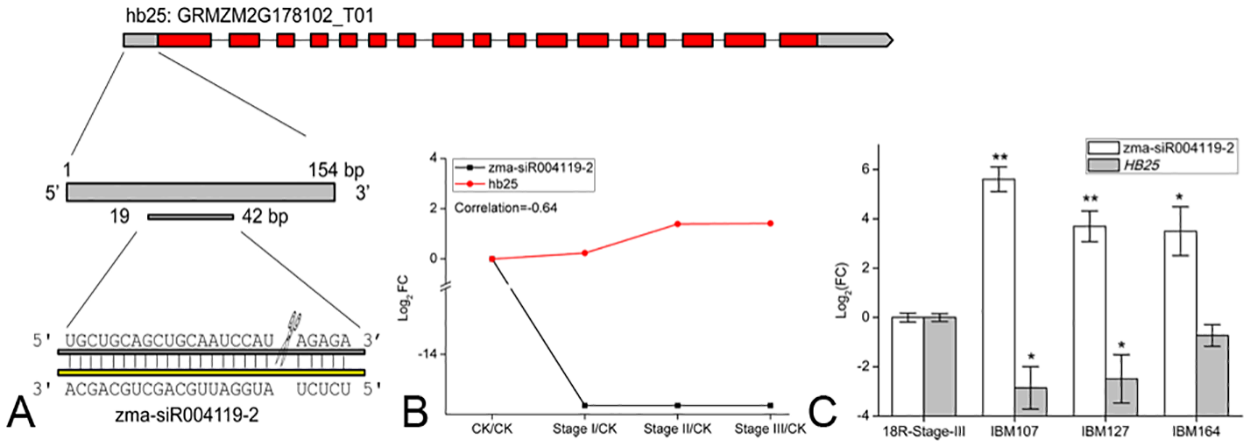
*HB25* was cleaved by zm-siR004119-2. (A) The cleavage site of *HB25* was in the 5’ UTR at 37–38 bp. (B) The relative expression levels of zm-siR004119-2 and *HB25* during callus formation. Expression is expressed as log_2_ (FC) as calculated in the comparison of the samples from Stage I, II and III to that of CK. (C) The relative expression levels of zm-siR004119-2 and *HB25* calli induced from the 18R, IBM107, IBM127 and IBM164 lines. Expression level is calculated for the comparison of the samples from IBM107, IBM127 and IBM164 to that from 18R, and the results are shown as the means ± SD (n = 3). *P < 0.05 (Student’s t-test) for the differences between 18R and IBM107, 18R and IBM127, or 18R and IBM164.

The 19–42 base pairs (bp) in the 5’ UTR region of GRMZM2G178102, which encodes Homeobox-transcription factor 25 (HB25), were bound by zma-siR004119-2 (Fig 5A). The expression level of zma-siR004119-2 decreased during Stages I, II and III both in the deep sequencing and real-time qPCR data. In contrast, *HB25* increased during Stages I, II and III. In addition, the correlation between zma-siR004119-2 and *HB25* was less than -0.64 (Fig 5B). To verify these results, we screened the tag of this gene using our previous degradome sequencing data. As predicted, the 1–38 bp portion of the 5’ region of *HB25* was degraded, which further demonstrated that zma-siR004119-2 can cleave the *HB25* gene at 37–38 bp (Fig 5A). To confirm that zma-siR004119-2 and *HB25* are involved in callus induction, we detected their expression levels in different types of calli induced from the 18R and 3 lines (i.e., IBM107, IBM127 and IBM164) with a low embryonic callus formation rate. As expected, the expression levels of zma-siR004119-2 in IBM107, IBM127 and IBM164 were significantly higher than those in 18R (*P* < 0.05, student’s t-test), and the expression levels of *HB25* in IBM107, IBM127 and IBM164 were significantly lower than those in 18R. *HB25* is a homolog of AT2G34710 (identity = 61.4%, coverage = 87.7%), which encodes Homeobox Protein 14 (ATPHB14), a member of the Class III HD-Zip genes. ATPHB14 have been shown to control the establishment of polarity in lateral organs in plants [37]. Furthermore, the *atphb* mutant showed a defect in auxin polar transport by influencing the expression of *PIN1* [38]. As discussed above, auxin is important for callus induction, and the up-regulation of *HB25* suggests that this gene functions directly or indirectly in promoting the expression of the *PIN1* genes. To verify this hypothesis, we analyzed our previous RNA-seq data and found that *PIN1* is increased during all callus induction stages [2].

**Fig 6:**
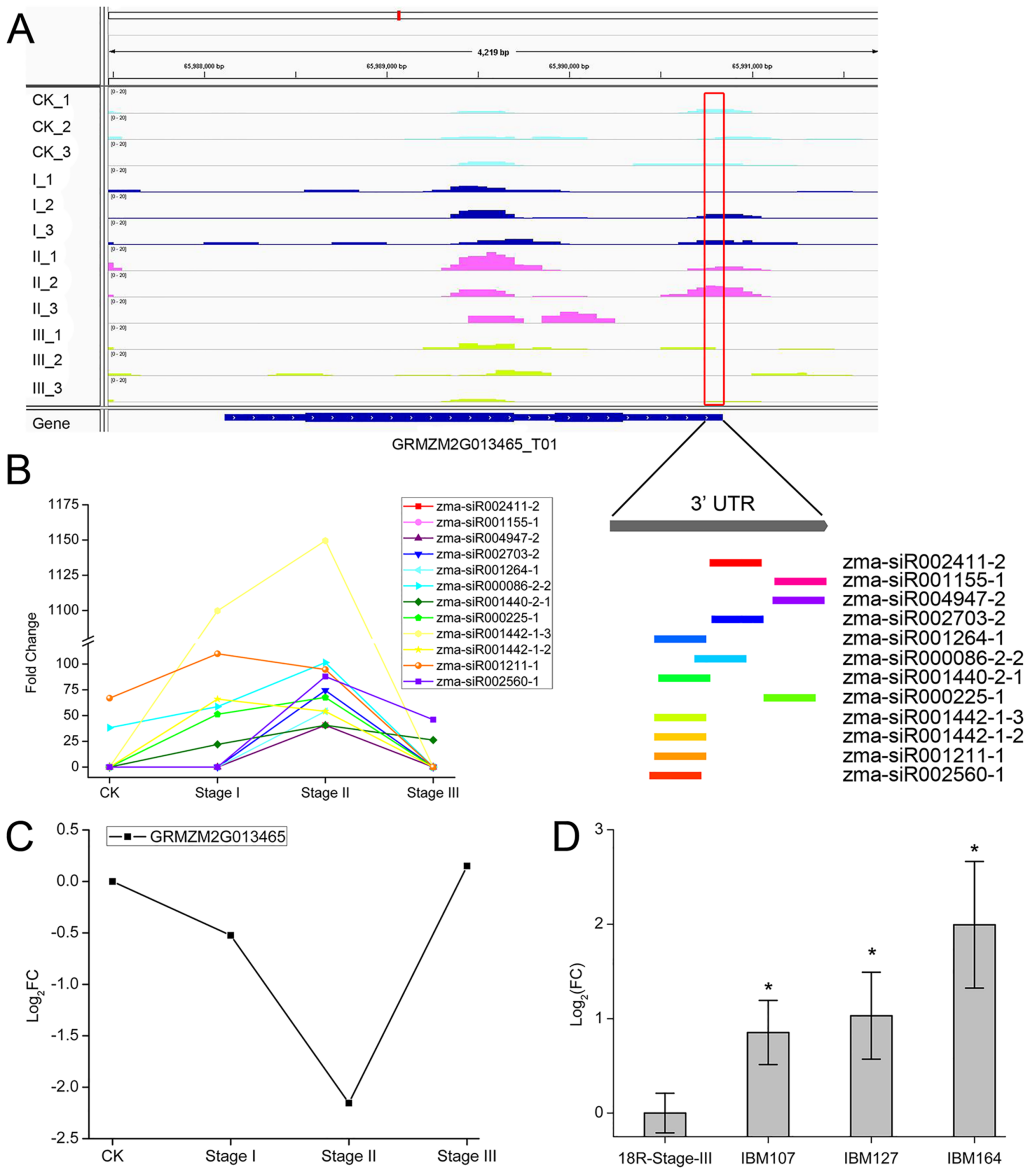
Associations among siRNA up-regulation, DNA hyper-methylation and transcriptional down-regulation of GRMZM2G013465 in CK and samples from the 3 stages. (A) IGV track of the MeDIP data in GRMZM2G013465. Turquoise, blue, pink and yellow: MeDIP-seq tracks of the samples from the CK group and samples from Stages I, II and III, respectively; red outlines the hyper-methylated region of the gene of interest. The colored rectangles represent the differentially expressed siRNAs targeting the 3’ UTR of GRMZM2G013465. (B) Fold changes of these differentially expressed siRNAs during callus induction. (C) Fold changes of GRMZM2G013465 in the 3 samples calculated from the deep sequencing and real-time qPCR data. (D) The expression levels of GRMZM2G013465 in different types of calli induced from the 18R, IBM107, IBM127 and IBM164 lines. Expression is calculated for the comparison of samples from IBM107, IBM127 and IBM164 to that from 18R, and the results are shown as the means ± SD (n = 3). *P < 0.05 and ** P < 0.01 (Student’s t-test) for differences between 18R and IBM107, 18R and IBM127, or 18R and IBM164.

In addition, GRMZM2G013465 was annotated as a protein phosphatase type 2A regulator that is involved in signal transduction. Interestingly, 12 24-nt DE-siRNAs target the 3’ UTR of this gene (Fig 6A). The expression levels of these 24-nt DE-siRNAs increased, showing an opposite expression pattern to that of this gene (Fig 6B and 6C). Moreover, the expression levels of GRMZM2G013465 in the calli (induced from IBM107, IBM127 and IBM164) were significantly higher than those induced from 18R (Fig 6D). In addition, it has been reported that 24-nt siRNAs can trigger DNA methylation [23]. We further analyzed the methylation level of this gene. As shown in Fig 6A, during Stages I and II, the 3’ UTR of this gene was hyper-methylated, resulting in the down-regulation of GRMZM2G013465, which further influenced the expression of the downstream genes.

## Conclusion

We identified 861 DE-siRNAs and 576 corresponding differentially expressed target genes. Based on the cluster analysis and GO function enrichment analysis, these target genes were classified into 3 clusters, and most of these genes function in controlling metalloexopeptidase activity, catalase activity, transcription regulation, and O-methyltransferase activity. Among these differentially expressed siRNAs, zma-siR001849-2, zma-siR001071-1, zma-siR003715-1-1, zma-siR004720-2, zma-siR002558-2, zma-siR000403-2, and zma-siR002521-1 target *PLT5-like*, *ARF15*, *SAUR-like*, *FAS1-like*, *Fea3*, *SCL5*, and *Zmwox2A*, respectively. These genes also showed a significantly changed expression (*FDR* < 0.001) during callus induction and were previously reported to function in controlling auxin transportation, stem cell development, and meristem development. Homeobox-transcription factor 25 was identified as the target gene of zma-siR004119-2, which was confirmed by our degradome sequencing data. Twelve 24-nt siRNAs were identified to target the 3’ UTR of GRMZM2G013465, leading to DNA methylation near the target site and further resulting in decreased expression.

## Supporting information

S1 Fig The correlation between the deep sequencing and RT-qPCR data during Stages I, II and III. The Expression level was calculated for the comparison of the samples from Stages I, II and III to that from CK.
(TIF)

S1 Table Basic information regarding the siRNAs and their target genes. (XLSX)

S2 Table The correlation and *P*-*value* among the siRNAs and the expression levels and methylation levels of the target genes. (XLSX)

S3 Table The primers used for the real-time qPCR of the siRNAs and target genes. (XLSX)
