# Supplementary figures and images for: Endogenous small interfering RNAs associated with maize embryonic callus formation
Source: PLoS One. 2017 Jul 3;12(7):e0180567. doi: 10.1371/journal.pone.0180567 (PMC5495461; doi:10.1371/journal.pone.0180567)

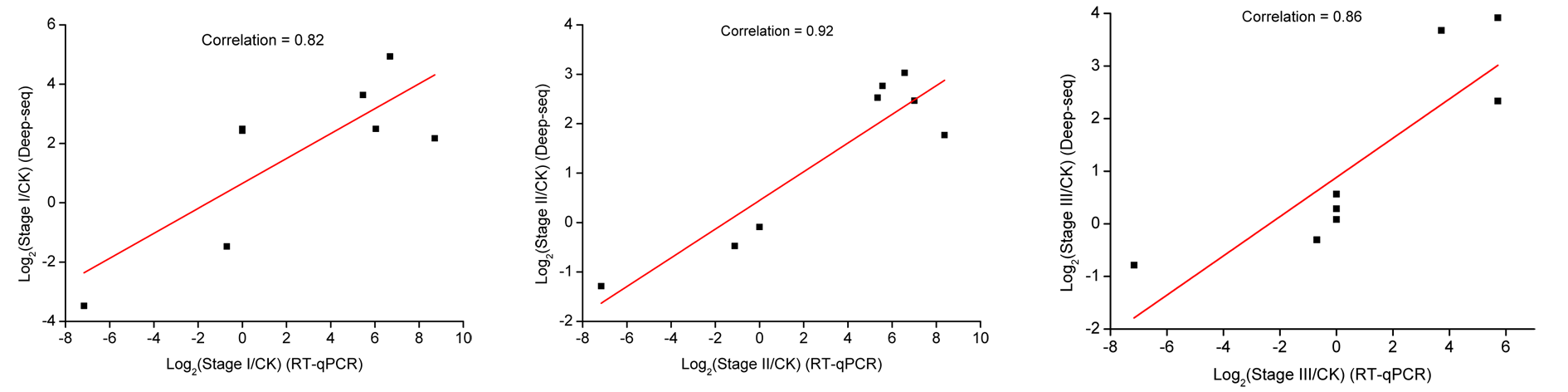

Supplement: S1 Fig — The Expression level was calculated for the comparison of the samples from Stages I, II and III to that from CK. (TIF) [file pone.0180567.s001.tif]
